# Mucosal melanoma of hard palate

**DOI:** 10.4322/acr.2024.522

**Published:** 2024-11-14

**Authors:** Zahed Ali Qamer, Monika Maharjan, Kranthi Kumar Jandrasupalli, Bommisetty Lokesh, Amit Tyagi, Ravi Hari Phulware

**Affiliations:** 1 All India Institute of Medical Sciences, Department of Pathology & Laboratory Medicine, Rishikesh, Uttarakhand, India; 2 All India Institute of Medical Sciences, Department of Radiodiagnosis, Rishikesh, Uttarakhand, India; 3 All India Institute of Medical Sciences, Department of Otolaryngology and Head and Neck Surgery, Rishikesh, Uttarakhand, India

**Keywords:** Melanoma, Malignant, Palate, Hard, Cancer, Mucous Membrane

## Abstract

Melanoma arising in the hard palate is an exceedingly rare entity, comprising a minute fraction of all melanoma cases. The absence of specific clinical signs often leads to delayed diagnosis and subsequent challenges in treatment planning. We discussed the existing literature to elucidate the epidemiology, risk factors, and molecular pathways associated with melanoma of the hard palate. Additionally, we discuss the importance of a multidisciplinary approach involving dermatologists, otolaryngologists, oncologists, and pathologists in diagnosing and managing this condition. A 62-year-old male patient presented with a pigmented lesion on the hard palate mucosa, which was initially asymptomatic but gradually increased in size. Biopsy revealed melanoma, confirmed through immunohistochemical staining. Staging investigations indicated a metastatic disease. Surgery followed by adjuvant therapy was planned; however, he was lost for the follow-up. Melanoma originating from the hard palate mucosa is exceedingly rare, posing diagnostic and therapeutic challenges. Early detection, accurate diagnosis, and prompt multidisciplinary management are crucial for optimal outcomes. This case underscores the importance of comprehensive evaluation and tailored treatment strategies in patients with uncommon mucosal melanomas.

## INTRODUCTION

Primary oral mucosal melanoma (OMM) is a rare malignancy that arises from melanocytes located within the basal layer of the oral mucosal epithelium among the basal keratinocytes, with an annual incidence of 1.2 cases per 10 million.^1^ This neoplasm exhibits a higher prevalence among Japanese, African, and Hispanic individuals, while its occurrence within the Indian subcontinent is exceedingly rare.^2^ It accounts for 1-2% of all oral malignancies, and its incidence rate is reported to be 0.2 to 8% of all melanomas in the Western population. However, the data on the Indian population is not clear.^2^ This malignancy predominantly affects individuals aged 40 to 70 years, with a notable rarity in those under 30 years, and exhibits a slight predilection for males.^3^ In females, the mucosa of the external genitalia is the most common site where mucosal melanoma (MM) is seen.^2,3^ The hard palate (47%) and gingival mucosa (27.6%) are the most affected sites, with lesser occurrences observed in the retromolar region and buccal mucosa.^1,2^ Although chronic trauma, tobacco use, and environmental factors have been suggested as potential contributors to disease development, direct causative links remain elusive. Surgical excision serves as the preferred treatment modality, often supplemented by adjuvant radiotherapy or chemotherapy.^3^ Although mucosal melanoma represents only a small fraction of all melanoma cases, it carries a worse prognosis compared to cutaneous melanoma.^4^ Moreover, the prognosis is worse with common nodal and distant metastasis. 5-year survival rate ranges from 25 to 40%.^5,6^

We report a rare case of primary oral mucosal melanoma of the hard palate presenting with widespread metastasis to lung and regional lymph nodes.

## CASE REPORT

**Figure 1:**
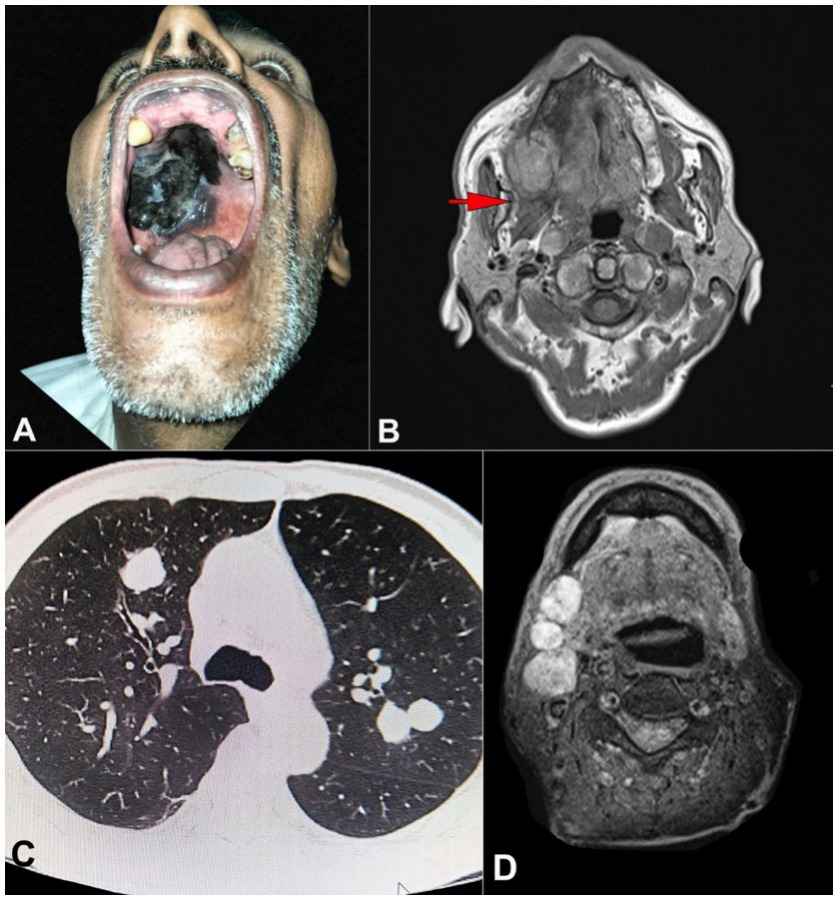
**A –** Gross view of the oral examination shows a blackish mass in the hard palate; **B –** Contrast-enhanced magnetic resonance imaging (CE-MRI) shows a well-defined irregularly shaped T2 hypointense lesion (3.7x3.2x2.0 cm) at the right side of hard palate; **C –** Axial thoracic HRCT, lung window image, showing - well defined round soft tissue attenuation lesions are seen in the anterior segment of the right upper lobe, and the apical-posterior segment of the left upper lobe; **D** – T1 fat-saturated images show enlarged cervical lymph nodes in the right level Ib and II station.

A 62-year-old male presented with a chief complaint of a painless blackish discoloration within the oral cavity, persisting for approximately one year, with a recent rapid increase in size noted over the past four weeks. The patient, a laborer by profession, has a notable medical history characterized by chronic smoking spanning 30 years, chronic alcohol consumption for 20 years, and a concurrent diagnosis of type II diabetes mellitus managed pharmacologically. He presented with a complaint of dysphagia for the past four weeks and dyspnea persisting for the past year, without associated symptoms of cough, chest pain, fever, or any reported contact with tuberculosis-infected individuals or any long-term exposure to anti-tubercular drugs. Denying any history of any dental procedure in the past, the patient also reported no exposure to pigments, molds, or dust and lacked any tattoos across his body. On physical examination, pallor was evident, alongside bilateral cervical lymphadenopathy, while systemic assessment revealed no discernible abnormalities. On local examination, a prominent blackish proliferative lesion was observed, spanning the hard palate and extending posteriorly to the palatoglossal arch, concurrently involving the medial gingival mucosa of the upper right alveolus (Figure 1A). The lesion measured approximately 6 x 4.5 cm in dimensions. The lesion was nontender, fixed to underlying tissue, and did not bleed on touch. Cartridge Based Nucleic Acid Amplification Test (CB-NAAT/GeneXpert) test using sputum sample was negative for tuberculosis.

Following a high-resolution computed tomography (HRCT) scan of the chest, the patient presented with multifocal ground glass centrilobular opacities diffusely distributed throughout the bilateral lung fields, presumed to be metastatic deposits (Figure 1C). Subsequently, contrast-enhanced computed tomography (CECT) of the head and neck revealed an indistinct, heterogeneously enhancing soft tissue thickening localized to the right hard palate, with involvement of the gingival mucosa laterally, accompanied by a few enlarged hypo-enhancing lymph nodes in the right level IB and II regions (Figure 1D). A subsequent contrast-enhanced magnetic resonance imaging (MRI) of the head and neck delineated a well-circumscribed irregular lesion within the right hard palate (Figure 1B). This lesion exhibited T2/STIR hypointensity with areas of T1 hyperintensity and manifested heterogeneous enhancement following contrast administration.

The lateral extension was noted into the alveolar process of the right hemimaxilla, with extension to the gingivobuccal sulcus and superiorly abutting and eroding the floor of the right maxillary sinus.

**Figure 2:**
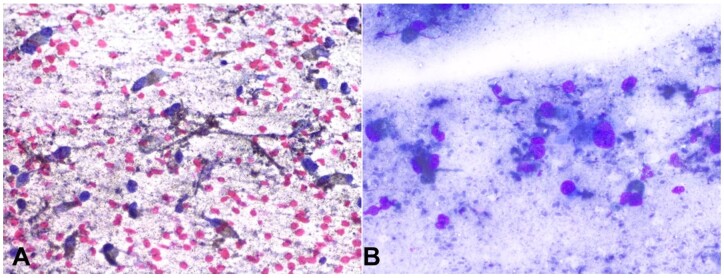
Photomicrographs of the fine needle aspiration cytology. **A** – Papanicolaou stained aspirate smear from the lesion preparations shows discohesive malignant epithelioid to spindle cells with delicate, pigmented cytoplasm in a background of dispersed and finely granular melanin pigment (PAP x200); **B –** May Grunwald-Giemsa stain (MGG) smear shows large atypical cells with dispersed and finely granular melanin pigment obscuring other cytological details (MGG x400).

As an institutional practice, we perform intraoral FNACs of intraoral masses not suspected of squamous cell carcinoma. Hence, the FNAC of the intraoral mass was compared with the FNAC of metastatic cervical lymph nodes to confirm the primary lesion. An intraoral fine-needle aspiration cytology (FNAC) procedure was conducted on the lesion using a 25-gauze needle and a 5ml syringe, revealing the presence of atypical cells predominantly distributed singly and in small clusters. These cells exhibited notable nuclear pleomorphism, characterized by varied nuclear sizes and shapes, and a prominent nucleocytoplasmic ratio. Additionally, there was evidence of nuclear hyperchromasia and conspicuous nucleoli. The cytoplasm of individual cells displayed a moderate volume and contained coarse brownish granules, as visualized upon Papanicolaou (PAP) staining (Figure 2A). This brownish pigment was also observed scattered throughout the background, in conjunction with the presence of blood and inflammatory cells (Figure 2B).

Fine-needle aspiration cytology of the right level III cervical lymph node further corroborated the presence of atypical cells exhibiting the aforementioned morphological characteristics within a lymphoid background, thus confirming the diagnosis of lymph nodal metastasis.

**Figure 3:**
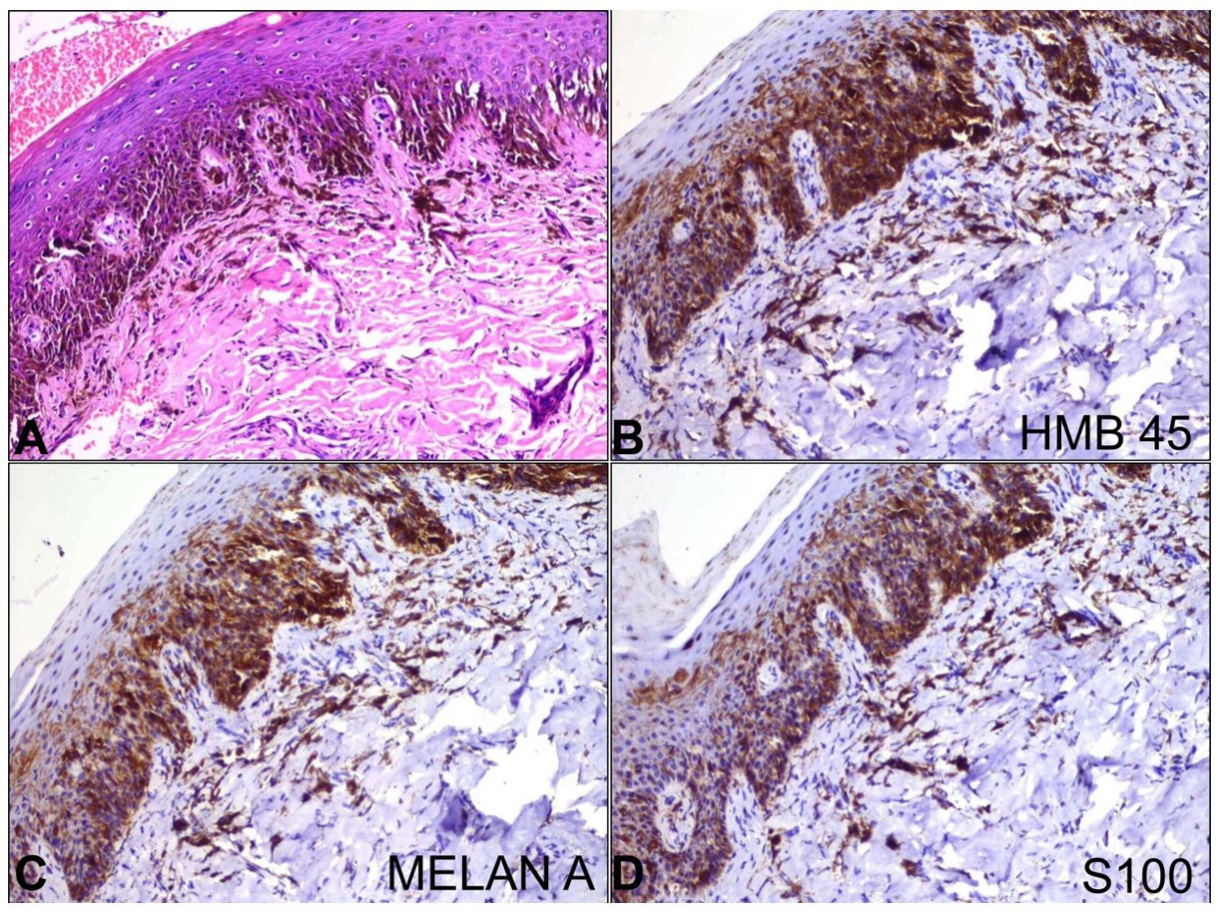
Photomicrograph of the biopsy. **A** – demonstrates hyperplastic stratified squamous epithelium with pagetoid infiltration as well as upper lamina propria involvement caused by pleomorphic spindle to epithelioid melanocyte cells (H&E, x200); On Immunohistochemistry these tumor cells are positive for **B** – HMB-45 (B x200), **C** – Melan A (C x200) and **D** – S-100 protein (D x200).

Concurrently, an incisional biopsy was performed on the hard palate lesion and a histopathological examination was performed. Microscopic evaluation revealed intact mucosal and submucosal layers, with heavily pigmented basal keratinocytes and notable proliferation of melanocytes observed as individual cells and nests, predominantly involving the subepithelial regions (Figure 3A). These cells exhibited enlarged epithelioid morphology with abundant eosinophilic cytoplasm, marked by heightened intracellular dark brownish coarse pigmentation. The pigmentation was so dense that the morphology of tumor cells was obscured. Notably, cytological atypia was observed in some cells, characterized by a high nucleocytoplasmic ratio and prominent nucleoli. Angioinvasion or neural invasion was not noted. Immunohistochemical analysis with adequate controls demonstrated positivity for Human Melanoma Black (HMB45) (Figure 3B), Melan A (Figure 3C), and S100 (Figure 3D), confirming the melanocytic origin of the lesion. The patient lacked any melanocytic lesions elsewhere, thus effectively ruling out metastasis. These histo-morphological and immunohistochemical findings collectively supported a primary oral mucosal melanoma diagnosis.

The treatment plan for this OMM was total removal of the tumor with at least a 1-cm margin, followed by radiotherapy plus chemotherapy. Medical information was provided to the patient and his family regarding the diagnosis, staging, therapeutic options, and prognosis. The patient and his family members said that they would consider seriously whether the patient could tolerate the surgery. Surgery was planned for the patient, but he was lost for the follow-up.

## DISCUSSION

MM represents a subset of rare neoplastic entities characterized by their occurrence within mucosal linings.^5,6^ These malignancies manifest predominantly within anatomical sites such as the nasal cavity and paranasal sinuses, traversing the entirety of the gastrointestinal tract from the oral cavity to the anorectum, as well as along the genitourinary tract, with notable predilection for regions such as the vulvar and vaginal mucosa. Additionally, MM may manifest within the tarsal conjunctiva.^6,7^

MM exhibits a distinct pathogenesis influenced by genetic, environmental, and immunological factors. Unlike cutaneous melanoma (CM), MM lacks identifiable risk factors.^6^ In addition to cigarette smoking, denture irritation, and alcohol consumption, chronic infections caused by microorganisms and mechanical stress from routine activities may influence tumorigenesis in the mucosal membrane.^7,8^ Unlike CM, ultraviolet radiation exposure plays a lesser role in MM development due to the shielding of mucosal membranes.^9^ Chronic inflammation and irritation of mucosal tissues, along with the presence of precursor lesions like mucosal melanosis, contribute to malignant transformation.^8^ Dysregulation of molecular signaling pathways and interactions within the mucosal immune microenvironment further contributes to tumor progression.^9,10^ Understanding these multifaceted mechanisms is crucial for developing targeted therapies and improving outcomes for patients with mucosal melanomas.

Distinguishing primary OMM from metastatic oral melanoma involves evaluating clinical history, histopathology, immunohistochemistry, molecular analysis, and radiological imaging. Primary MM typically presents as solitary lesions without prior CM, exhibiting in situ components and potentially distinct genetic mutations. Metastatic oral melanomas arise from disseminated disease, lacking in situ components and often manifesting with primary lesions elsewhere in the body.^7,8^

Primary OMM typically manifests as asymptomatic, irregularly shaped macular lesions in black-brown or tan hues. Alternatively, they may present appear blue or purple due to deeper pigmentation. While less than 2% of all melanomas are non-pigmented or amelanotic, the incidence of amelanotic melanomas is significantly higher in the oral mucosa, up to two-thirds.^5,7^ Amelanotic variants can appear dark red or pink. This variation in presentation underscores the importance of thorough histological and immunohistochemical evaluation to accurately identify and diagnose MM in the oral cavity.^8,9^ As the disease progresses, the lesions can ulcerate, become erythematous and nodular, and invade deeper tissues, leading to pain. The onset of pain is often associated with tumor growth, ulceration, and secondary infection.^3,4^

In contradistinction to CM, MM exhibits a discernibly diminished burden of mutational load, characterized by recurrent copy number aberrations and structural genomic rearrangements. However, there is a higher prevalence of *KIT* mutations in MM.^5,6^ Noteworthy is the conspicuous absence of *BRAF* mutations in MM. Approximately 20%–40% of cases evince KIT mutations, with *NRAS* mutations detected in 15%–20% of instances.^7^ Remarkably, distinct mutation spectra manifest across different mucosal sites; for instance, *NRAS* mutations predominate in up to 43% of vaginal melanomas, while *KIT* mutations are prevalent in 25% of anorectal melanomas.^2,3^ In a recent study done by Song et al.^7^ DNA sequencing revealed BAP1 missense mutations in 4 of 12 OMM patients, while immunohistochemical staining showed loss of nuclear BAP1 expression correlating with worse overall survival and increased distant metastasis, establishing it as an independent prognostic factor in OMM.^1,3^ BCL-2 expression is associated with improved prognostic outcomes in MM. Conversely, abnormal p53 protein expression and the absence of p16 protein expression are correlated with an unfavorable prognosis.^1,4^ These findings underscore the heterogeneity intrinsic to MM, hinting at the existence of discrete molecular subtypes. Efforts have been made to categorize MMs into subtypes based on their molecular profiles; however, conducting statistically significant studies remains challenging due to their rarity.^4,5^

Available data regarding the cytological features of oral mucosal melanomas are relatively minimal compared to other diagnostic methods. Although attempts have been made to utilize FNAC for diagnosis, its effectiveness is hindered by challenges such as sample adequacy, heterogeneity of cell population, interpretation variability, and difficulty in performing intra-orally.^7,9^ Cytological characteristics indicative of melanocytic origin include the presence of atypical melanocytes showing enlarged nuclei, prominent nucleoli, high nuclear-to-cytoplasmic ratio, irregular nuclear contours, and cytoplasmic melanin pigment. Additionally, the identification of multinucleation, mitotic figures, and cellular pleomorphism may support the diagnosis of OMM.^5,6^

The definitive diagnosis of OMM is achieved by histopathological examination of a biopsy specimen obtained from the lesion, followed by confirmation through IHC. The 1997 WESTOP Banff Workshop^6^ classified OMMs into three main types: in situ, invasive, and combined. In the in-situ pattern (15%), the neoplasm is limited to the mucosal epithelium, and in the invasive pattern (30%), it is found to infiltrate the connective tissue below the mucosal layer. A combination of these patterns is found in approximately 55% of the cases and is associated with advanced lesions.^4,6^ Neoplastic cells in OMM display a diverse array of morphologies, encompassing spindle-shaped, plasmacytoid, and epithelioid forms. Microscopic patterns comprise sheet-like, organoid, pseudo-alveolar, neurotropic, and desmoplastic. The nuclei are pleomorphic and hyperchromatic and usually contain one or more amphophilic nucleoli. In most cases, variable quantities of melanin are discernible, either within tumor cells or macrophages, or in extracellular particle configurations.^1,3^ Sometimes, there might be a complete absence of pigment where the diagnosis can be made solely on IHC. Tumor-associated stromal desmoplasia and chronic lymphocyte-dominated inflammatory infiltrates are frequently observed, distinguishing characteristics absent in melanocytic nevi. The presence of tumor-infiltrating lymphocytes is a favorable prognostic marker, whereas their absence is associated with lymph node metastasis and a less favorable prognosis configuration.^2,3^

Confirmation of the histopathological findings is done by IHC, where the tumor cells are strongly positive for S100 and HMB45 in almost all cases. Melan-A shows variable positivity in tumor cells. The cells show high proliferation activity with Ki67, which ranges from 15-60%.^3,4^ Additionally, Fatty Acid Synthase (FASN) expression aids in distinguishing oral melanomas from oral melanocytic nevi, as it demonstrates robust expression in mucosal surface melanomas.^11^ The similar color of melanin and commonly used chromogens create difficulty in interpretating IHC in cases of heavily pigmented lesions. Melanin bleaching by warm Hydrogen peroxide is very useful in such cases.^5,7^

Unlike CM, MM lacks histologic landmarks such as the granular layer and subcutaneous tissue, rendering standard staging systems like Clark and Breslow inapplicable. Patients with MM in the head and neck region are typically staged using the American Joint Committee on Cancer (AJCC) staging system.^8,9^ Alternatively, they can be divided into three stages: stage I, where the cancer is confined to the primary site; stage II, involving positive cervical lymph nodes; and stage III, marked by distant metastases. Within stage I tumors, the depth of invasion provides valuable prognostic information. In a study conducted by Prasad et al.^8^, involving 61 patients with stage I mucosal melanomas, the primary site and invasion depth emerged as significant predictors of cause-specific survival, with invasion depth being the sole parameter significantly impacting survival outcomes. In addition to the clinical stage, tumor thickness exceeding 5 mm, vascular invasion observed through light microscopy, and the emergence of distant metastases were identified as independent prognostic factors in a study by Patel et al.^9^

Once the diagnosis of OMM is confirmed, a whole-body PET scan and computed tomography are typically recommended as they are instrumental in differentiating primary disease from a clinically occult metastatic disease.^10^ Additionally, comprehensive tumor staging is crucial for head and neck contrast-enhanced CT, chest radiography, and liver function tests (to detect elevated aspartate aminotransferase/alanine aminotransferase). As lung metastasis is usually seen in OMM, the thoracic CT becomes the most important test in radiological surveillance in such patients. The application of fluorodeoxyglucose (FDG)-PET/CT in managing cutaneous melanoma is well-documented. However, its utility in MM remains underexplored. Given the high metabolic activity of MM, FDG-PET/CT is expected to offer comparable staging insights, though this hypothesis necessitates validation through large-scale clinical trials.^3,5^

Metastasis in MM poses a significant clinical challenge due to its aggressive behavior and limited therapeutic options. In primary OMMs, approximately 60% of patients demonstrate cervical lymph node involvement upon presentation. Distant metastases occur in 10% to 50% of cases at diagnosis, with the lungs being the most common site, followed by the liver, bones, and brain.^5,8^ The tendency for early dissemination through the lymphatic and blood vessels contributes to the disease's advanced stage at diagnosis and the high incidence of distant metastases among patients with mucosal melanoma.^8.9^

Due to its rarity, an optimal treatment modality for OMM has yet to be established. The preferred approach is complete tumor resection with clear margins.^2^ However, en bloc resection with clear margins is rarely feasible for oral mucosal melanoma due to the intricate anatomical structure of the oral and maxillofacial region.^3,4^ MM is generally regarded as relatively radioresistant. Although radiotherapy (RT) can be utilized as adjuvant treatment or for palliative care, it is not typically the primary treatment modality due to the tumor's limited response, and its impact on survival remains insufficiently investigated. In a study done by Naganawa et al.^10^ Carbon-ion RT was found to be an effective treatment option with acceptable toxicity for OMM.^6,8^ Chemotherapy utilizing platinum analogs, nitrosoureas, dacarbazine, and immunotherapy involving IL-2 have demonstrated limited efficacy in terms of treatment response. To date, only a limited number of targeted therapies for OMM have been evaluated in clinical trials, including *BRAF*, *MEK*, CDK4/6, and C-KIT inhibitors. However, they have limited clinical use and efficacy. The recurrence rate is found to be 20% even after complete surgical excision with negative margins.^3,5^

## CONCLUSION

This case has been reported for its rarity, diagnostic and treatment challenges, and for highlighting the cytological and histopathological findings. As this type of tumor usually presents at an advanced stage, regular surveillance by dentists and other oral physicians is necessary to pick up these cases at an early stage where appropriate treatment can be given.
